# Deficient invariant natural killer T cells had impaired regulation on osteoclastogenesis in myeloma bone disease

**DOI:** 10.1111/jcmm.13554

**Published:** 2018-02-23

**Authors:** Fengjuan Jiang, Hui Liu, Zhaoyun Liu, Siyang Yan, Jin Chen, Qing Shao, Lijuan Li, Jia Song, Guojin Wang, Zonghong Shao, Rong Fu

**Affiliations:** ^1^ Department of Graduate School Tianjin Medical University Tianjin China; ^2^ Department of Hematology Tianjin Medical University General Hospital Tianjin China

**Keywords:** α‐galactosylceramide, invariant natural killer T cell, myeloma bone disease, osteoclastogenesis

## Abstract

Recent research showed that invariant natural killer T (iNKT) cells take part in the regulation of osteoclastogenesis. While the role of iNKT cells in myeloma bone disease (MBD) remains unclear. In our study, the quantity of iNKT cells and the levels of cytokines produced by them were measured by flow cytometry. iNKT cells and osteoclasts were induced from peripheral blood mononuclear cells after activation by α‐GalCer or RANKL in vitro. Then, gene expressions and the levels of cytokines were determined by RT‐PCR and ELISA, respectively. The results showed that the quantity of iNKT and production of IFN‐γ by iNKT cells were significantly decreased in newly diagnosed MM (NDMM), and both negatively related with severity of bone disease. Then, the osteoclasts from healthy controls were cultured in vitro and were found to be down‐regulated after α‐GalCer‐stimulated, while there was no significant change with or without α‐GalCer in NDMM patients, indicating that the regulation of osteoclastogenesis by iNKT cells was impaired. Furthermore, the inhibition of osteoclastogenesis by iNKT cells was regulated by IFN‐γ production, which down‐regulated osteoclast‐associated genes. In conclusion, the role of α‐GalCer‐stimulated iNKT cells in regulation of osteoclastogenesis was impaired in MBD, as a result of iNKT cell dysfunction.

## 1 INTRODUCTION

Multiple myeloma (MM) is a haematologic malignancy on account of the uncontrolled proliferation of plasma cells in the bone marrow (BM). Myeloma bone disease (MBD) is the most common complication of MM, with up to 70% of them having osteolytic lesions at diagnosis.^1^ Bone lesions in MM are induced by imbalance between osteoclasts (OCs) and osteoblasts (OBs), the activation of OCs and inhibition of OBs. Many researches focused on the function of OCs and found OCs play a critical role in the pathogenesis of MBD.^2^ As we all know, osteoclastogenesis and activation of bone resorption are regulated by the RANK/RANKL/OPG axis.^3^ Immune cells, such as T cells, express RANKL, which contributed to the pathogenesis of MBD.^4^, ^5^, ^6^ While T cells also resist osteoclastogenesis by producing interferon‐γ (IFN‐γ), which counterbalance the action of RANKL.^4^, ^7^, ^8^ Meanwhile, several cytokines are involved in osteoclastogenesis, such as interleukin‐17 (IL‐17), produced by Th17 cells, is shown to active OCs and induce bone resorption,^9^ whereas IL‐4 produced by B, Th1 and Th2 cells has an antiosteoclastogenic effect.^10^, ^11^

Invariant NKT (iNKT) cells are a distinct subset of T cells that express a semi‐invariant TCR α‐chain encoded by Vα24‐Jα18 paired with a limited repertoire of Vβ chains encoded by Vβ11 in human beings and recognize glycolipid ligands presented by the non‐polymorphic MHC class I‐like molecule CD1d.^12^, ^13^ iNKT cells can be activated by a synthetic glycolipid isolated from a marine sponge, α‐galactosylceramide (α‐GalCer or KRN7000), leading to rapid production of several cytokines such as IL‐4 and IFN‐γ.^14^, ^15^ NKT cells exhibit bridges connecting the innate immune system to the adaptive immune system. Furthermore, they are able to interact with immune cells, such as monocytes, T cells, B cells, dendritic cells and NK cells, by producing Th1 and Th2 cytokines.^16^

Many studies have confirmed that NKT cell level and function are reversibly defective in MM.^17^, ^18^, ^19^, ^20^ In addition, a recent study showed that the function of iNKT is defective which is related to osteoclastogenesis and inflammatory bone destruction in RA patients.^21^ Furthermore, a previous study illustrated that iNKT cells have the unique characteristic of enhancing osteoclast progenitor and precursor development.^22^ However, the effect of iNKT on MBD remains unknown. The aim of this study was to investigate the role of iNKT cells during osteoclastogenesis and the underlying mechanism of osteoclastogenesis dysregulation in MBD.

## 2 MATERIALS AND METHODS

### 2.1 Patients and sample

The study cohort included 37 newly diagnosed MM (NDMM) patients, 21 remission MM patients (including very good partial response (VGPR), complete remission (CR) or stringent complete response [sCR]), 8 relapsed/refractory MM (RMM) patients and 23 age‐ and sex‐matched healthy controls (HCs; Age: median, 58 years; range, 44‐74 years; Gender: 14 males, 9 females). All the patients were inpatients in the Hematology Department of Tianjin Medical University General Hospital from October 2015 to May 2017 and diagnosed and assessed after treatment according to International Myeloma Working Group uniform response criteria.^20^ According to body X‐ray scanning data obtained before treatment within 1 week after diagnosis, bone morbidity was graded into three stages (Stage A included patients with no osteolytic lesions or osteoporosis alone; Stage B included patients with one to three osteolytic lesions, and Stage C included patients with more than three osteolytic lesions and/or a pathological fracture).^23^, ^24^ We have used the <3/>3 as cut‐off for bone lesions as advanced bone disease (BD; Stage C) includes more than three lytic lesions in the Durie‐Salmon staging system. The clinical characteristics of the patients are shown in Table 1. In our study, remission MM patients have received bortezomib‐based regimens (including bortezomib and dexamethasone (VD); bortezomib, cyclophosphamide and dexamethasone (VCD); bortezomib, thalidomide and dexamethasone (VTD) at least 4 cycles). Relapsed/refractory patients have received at least triplet regimens including bortezomib and thalidomide (lenalidomide) or combination with doxorubicin/cisplatin/etoposide. This study was approved by the Ethical Committee of the Tianjin Medical University. Written informed consent was obtained from all participants. Peripheral blood mononuclear cells (PBMCs) were isolated from peripheral venous blood of patients and HCs mentioned above using Ficoll‐Paque Plus solution (Amersham Biosciences).

**Table 1:** Patients’ base‐line characteristics

	Newly diagnosed MM patients (n = 37), %	Remission MM patients (n = 21), %	Relapsed/refractory MM patients (n = 8), %
Gender			
Male	22 (59.5)	12 (57.1)	5 (62.5)
Female	15 (40.5)	9 (42.9)	3 (37.5)
Age (median)	62	61	63
Range	45~77	47~74	59~76
ISS stage			
I	2 (5.4)	3 (14.3)	1 (12.5)
II	13 (35.1)	6 (28.6)	1 (12.5)
III	22 (59.5)	12 (57.1)	6 (75.0)
M‐component type			
IgG	13 (35.1)	6 (28.6)	4 (50.0)
IgA	11 (29.8)	4 (19.0)	1 (12.5)
IgM	1 (2.7)	0 (0)	0 (0)
Light chain only	9 (24.3)	9 (42.9)	3 (37.5)
Non‐secretory	3 (8.1)	2 (9.5)	0 (0)
β_2_‐microglobulin (mg/L)			
<5.5	14 (37.8)	16 (76.2)	1 (12.5)
≥5.5	23 (62.2)	5 (23.8)	7 (87.5)
Hb (g/L)			
<100	27 (73.0)	4 (19.0)	6 (75.0)
≥100	10 (27.0)	17 (81.0)	2 (25.0)
Ca (mmol/L)			
>2.75	15 (40.5)	0 (0)	1 (12.5)
≤2.75	22 (59.5)	21 (100.0)	7 (87.5)
Serum creatinine (μmol/L)			
<177	24 (64.9)	15 (71.4)	3 (37.5)
≥177	13 (35.1)	6 (28.6)	5 (62.5)
Bone disease stage			
Stage A	8 (21.6)	8 (38.1)	3 (37.5)
Stage B	12 (32.4)	9 (42.9)	3 (37.5)
Stage C	17 (46.0)	4 (19.0)	2 (25.0)

MM, multiple myeloma; ISS, International Staging System; Hb, haemoglobin.

### 2.2 Flow cytometry

Invariant NKT cells were routinely identified phenotypically as Vα24‐positive and Vβ11‐positive T cells. Isolated PBMCs (1 × 10^6^ cells) were stained with 20 μL anti‐human Vα24 FITC (Beckman Coulter, Pasadena, CA), anti‐human Vβ11 PE (Beckman Coulter), anti‐human CD3 PerCP (Becton Dickinson, BD Biosciences, Franklin Lakes, NJ, USA) and their isotype control antibodies in the dark at 4°C for 15 minutes. Then, the cells were washed twice with PBS (phosphate balanced solution). At least 500 000 counts were obtained and analysed by Attune NxT acoustic focusing cytometer (Life Technologies) and CytExpert software (version 1.2).

CD34^+^OCN^+^ cells were routinely regarded as osteoblast precursors (OBPs),^25^ and CD14^+^CD16^+^ cells were routinely as osteoclast precursors (OCPs).^26^ Isolated PBMCs (1 × 10^6^ cells) were stained with 20 μL anti‐human CD34‐FITC (BD Biosciences), anti‐human OCN‐PE (R&D System, Abingdon, USA), anti‐human CD16‐FITC (BD Biosciences), anti‐human CD14‐PE (BD Biosciences) and their isotype control antibodies in the dark at 4°C for 30 minutes. Then, the cells were washed twice with PBS. At least 500 000 counts were obtained and analysed using a BD FACS Calibur cytometer and CellQuest software (version 6.0).

Cytokine expressions were detected by flow cytometry after stimulation with α‐GalCer in vitro as described.^27^ Isolated PBMCs (1 × 10^6^ cells/well) were incubated in RPMI 1640 medium supplemented with 10% foetal bovine serum, in the presence of α‐GalCer (200 ng/mL; BioVision Incorporated, Milpitas Boulevard, Milpitas, USA) or 0.1% DMSO (as control) in a 12‐well plate at 37°C in a humidified incubator containing 5% CO_2_. After 2 hours, brefeldin A (10 μg/mL; Sigma‐Aldrich) was included to stop exocytosis of cytokines. After an additional incubation time of 6 hours, the cells were stained with iNKT cells as described above, anti‐human CD8 APC‐Cy7 (BD Biosciences) and anti‐human CD4 PE‐Cy7 (BD Biosciences) in the dark at 4°C for 15 minutes and subsequently subjected to intracellular cytokine staining. The cells were then fixed in IntraSure Kit reagent A (BD Biosciences) for 5 minutes and permeabilized with IntraSure Kit reagent B (BD Biosciences) for 10 minutes at room temperature. Cells were incubated with 20 μL anti‐human IFN‐γ APC (BD Biosciences), anti‐human TNF APC (BD Biosciences), anti‐human IL‐4 APC (BD Biosciences), anti‐human IL‐13 APC (BD Biosciences) in the dark at 4°C for 30 minutes. Then, the cells were washed twice with PBS. At least 500 000 counts were obtained and analysed by Attune NxT acoustic focusing cytometer (Life Technologies) and CytExpert software (version 1.2).

### 2.3 iNKT cells culture induced by α‐GalCer in vitro

Isolated PBMCs were incubated in RPMI 1640 medium supplemented with 10% foetal bovine serum, 100 U/mL penicillin and 100 mg/mL streptomycin in the presence of IL‐2 (100 U/mL; PeproTech) and 200 ng/mL α‐GalCer or 0.1% DMSO (as control) and then seeded in a 24‐well plate at 1 × 10^6^ cells/well and cultured for 14 days at 37°C in a humidified incubator containing 5% CO_2_. Culture media with cytokines were replaced every 3 days. iNKT cells were detected as described above at day 0, day 7 and day 14 by flow cytometry (Attune NxT acoustic focusing cytometer, Life Technologies).

### 2.4 Culture of osteoclasts

Isolated PBMCs were incubated in α‐MEM medium supplemented with 10% foetal bovine serum, 100 U/mL penicillin and 100 mg/mL streptomycin in the presence of recombinant human RANKL (150 ng/mL; Miltenyi Biotec, USA), recombinant human macrophage colony‐stimulating factor (M‐CSF; 50 ng/mL; Miltenyi Biotec, USA) and α‐GalCer (200 ng/mL) or 0.1% DMSO (as control) and then seeded in a 24‐well plate at 1 × 10^6^ cells/well and cultured for 14 days at 37°C in a humidified incubator containing 5% CO_2_. Culture media with cytokines were replaced every 3 days. After culture, OCs were generated and used for subsequent experiments. A tartrate‐resistant acid phosphate (TRAP)‐staining kit was performed according to the manufacturer's instructions (Sigma‐Aldrich). TRAP‐positive multinucleated cells (≥3 nuclei) were defined as osteoclasts and their numbers were counted. To determine changes in osteoclastogenesis after cytokine blocking or intensifying, PBMCs (1 × 10^6^ cells/well) were cultured with RANKL (150 ng/mL), M‐CSF (50 ng/mL) and α‐GalCer (200 ng/mL) in the presence of cytokine or cytokine inhibitor (0.1% DMSO as control) for 14 days, then stained for TRAP. Cytokine‐blocking antibodies used were anti‐IFN‐γ (10 μg/mL) 21 (BD Biosciences) or recombinant IFN‐γ (10 ng/mL; BD Biosciences).

### 2.5 Quantitative real‐time PCR

Quantitative real‐time PCR was performed as described previously.^28^ RNA was extracted from OCs using the TRIzol reagent (Invitrogen, USA), and mRNA expression was quantified using the Bio‐Rad iQ 5 Real‐time system (Bio‐Rad, Hercules, CA, USA). The primer sequences of osteoclast‐associated genes are shown in Table 2.

**Table 2:** Primer sequences

Target	Sense and anti‐sense sequences	Base pairs (bp)
TRAP	F: 5′‐CCC GTT GGT GTT TAT GTG TG‐3′	120
	R: 5′‐CTA GGA TGG GTT GCG TGT CT‐3′	
OSCAR	F: 5′‐CCA GCT CTA GCG GGT ATC TG‐3′	128
	R: 5′‐CCA TGG CTT AGG GTG GTA TG‐3′	
RANKL	F: 5′‐CAG AGA AAG CGA TGG TGG AT‐3′	117
	R: 5′‐TAT GGG AAC CAG ATG GGA TG‐3′	
β‐actin	F: 5′‐TGG ACA TCC GCA AAG ACC TGT‐3′	160
	R: 5′‐CAC ACG GAG TAC TTG CGC TCA‐3′	

Bp, base pairs; TRAP, tartrate‐resistant acid phosphate; OSCAR, osteoclast‐associated receptor.

The SYBR Green (Invitrogen) was used as a double‐strand DNA‐specific dye. The amplification of TRAP utilized 40 cycles at 95°C for 30 seconds and 95°C for 5 seconds with the extension at 57.2°C for 30 seconds. The amplification of OSCAR utilized 45 cycles at 95°C for 30 seconds and 95°C for 5 seconds with the extension at 63.5°C for 30 seconds. The amplification of RANKL utilized 45 cycles at 95°C for 30 seconds and 95°C for 5 seconds with the extension at 57.5°C for 30 seconds. β‐actin was employed as the housekeeping gene to standardize the targeted mRNA expression. The levels of TRAP, OSCAR, RANKL were calculated using the 2^−∆∆Ct^ method [(Ct, target gene Ct, β‐actin)_sample_ − (Ct, target gene Ct, β‐actin)_control_] after normalizing the data according to the β‐actin mRNA expression.

### 2.6 Enzyme‐linked immunosorbent assay (ELISA)

OCs culture supernatants from the MM patients and HCs were harvested and stored at −80°C until time of analysis. The cytokine levels of IFN‐γ, TNF, IL‐4 or IL‐13 were measured by ELISA kit (R&D Systems) according to the directions of the manufacturer.

### 2.7 Electro chemiluminescence immunoassay (ECLIA)

Bone formation markers, osteocalcin (OCN) and procollagen I amino‐terminal propeptide (PINP), and bone resorption marker carboxy‐terminal cross‐linking telopeptide of type I collagen (CTX) were measured by ECLIA kit (Roche Diagnostics, Germany) performed with Elecsys Immunoassay Analyser 2010 according to the instructions of the manufacturer. We collected data from clinical laboratory of Tianjin Medical University General Hospital.

### 2.8 Statistical analyses

Student's *t* test was conducted for two‐group comparisons. For many‐group comparisons, one‐way ANOVA analysis or Kruskal‐Wallis test was used. Correlation between the different percentages of iNKT cells and all variables was determined by Spearman's correlation coefficient. The data are expressed as the mean ± SEM or median. Statistical analyses were performed using SPSS version 21.0 software. *P* values of <.05 were considered significant.

## 3 RESULTS

### 3.1 The quantity of iNKT cells reduced and was negatively related with bone disease in NDMM patients

**Figure 1:**
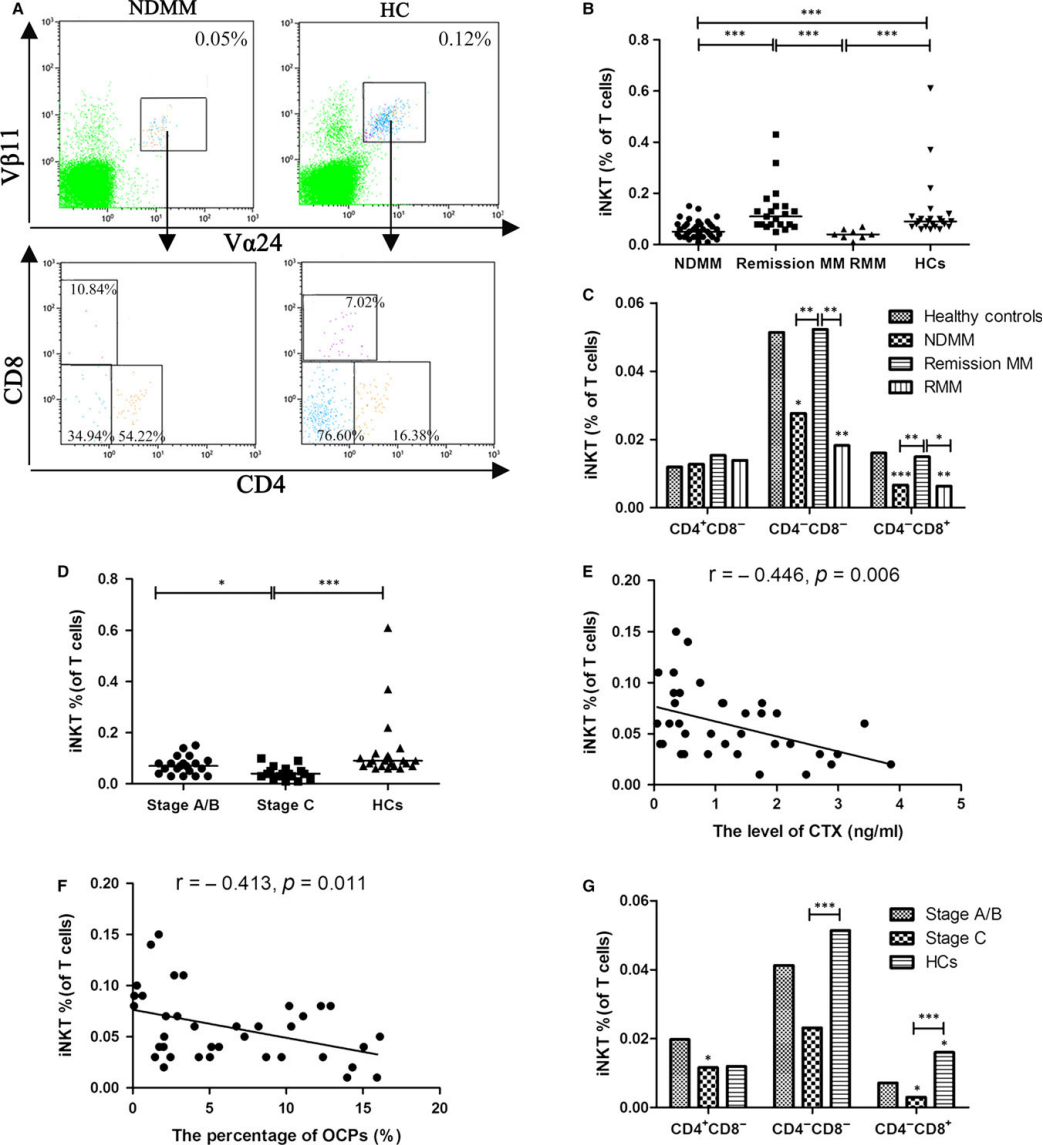
The level of iNKT cells frequency is reduced and significantly related with bone disease in newly diagnosed multiple myeloma (NDMM) patients. A, The percentages of Vα24^+^Vβ11^+^ T (iNKT) and CD4^+^, CD8^+^ and CD4^−^ CD8^−^ iNKT cell in T cells were examined by flow cytometry. B, The percentage of iNKT cells was significantly lower in patients with NDMM and relapsed/refractory MM (RMM) than that in healthy controls (HCs). The percentage of iNKT cells in MM patients achieved remission rised again compared with untreated NDMM patients. C, About the percentages of CD4^−^ CD8^+^ and CD4^−^ CD8^−^ (double‐negative, DN) iNKT cells of T cells, NDMM and RMM patients had significantly lower than HCs, while MM patients achieved remission had significantly higher than untreated NDMM patients. D, The percentage of iNKT cells was significantly lower in the patients with Stage C bone disease than those with Stage A/B bone disease and HCs. E, The percentage of iNKT cells was significantly correlated with the level of carboxy‐terminal cross‐linking telopeptide of type I collagen (CTX) respectively by Spearman's correlation coefficient. F, The percentages of iNKT cells were significantly correlated with the percentages of osteoclast precursors (OCPs) respectively by Spearman's correlation coefficient. G, The percentage of CD4^−^ CD8^+^ iNKT cells was significantly lower in the patients with Stage C bone disease than those with Stage A/B bone disease and HCs. (Medians of each group were compared using Kruskal‐Wallis test followed by all pairwise multiple comparisons; *P <* *.05, **P <* *.01, ***P <* *.001)

We analysed the percentages of iNKT cells in the T cell pool from peripheral blood of 37 NDMM patients, 21 remission MM patients, 8 relapsed/refractory MM patients and 23 age‐ and sex‐matched healthy controls by flow cytometry (Figure 1A). The percentage of Vα24^+^Vβ11^+^ T (iNKT) cells was significantly lower in patients with NDMM and RMM than that in HCs (median 0.05% and 0.04% vs 0.09%, *P *<* *.001 and *P *<* *.001, respectively). The percentage of iNKT cells in MM patients achieved remission rised again compared with untreated NDMM patients (median 0.11% vs 0.05%, *P *<* *.001; Figure 1B). According to the expression of CD4 and CD8, iNKT cells were subdivided into CD4^+^CD8^−^, CD4^−^CD8^+^ and CD4^−^CD8^−^ (double‐negative, DN) cell subsets (Table 3). About the percentages of CD4^−^CD8^+^ and DN iNKT cells of T cells, NDMM and RMM patients had significantly lower than HCs (for CD4^−^CD8^+^ iNKT cells, *P *<* *.001 and *P *=* *.008, for DN iNKT cells, *P *=* *.01 and *P *=* *.006, respectively), while MM patients achieved remission had significantly higher than untreated NDMM patients (*P *=* *.001 and *P *=* *.001, respectively; Figure 1C).

**Table 3:** The percentage of iNKT cells subsets in the T cell pool of NDMM patients, remission MM patients, RMM patients and HCs

Groups	Number	CD4^+^CD8^−^ iNKT (%)	CD4^−^CD8^−^ iNKT (%)	CD4^−^CD8^+^ iNKT (%)
HCs	23	0.0119 (0.0042‐0.0839)	0.0514 (0.0203‐0.5459)	0.0160 (0.0053‐0.0840)
NDMM	37	0.0127 (0.0026‐0.0962)	0.0276 (0.0042‐0.3464)	0.0066 (0.0008‐0.0306)
Remission MM	21	0.0153 (0.0056‐0.0621)	0.0523 (0.0185‐0.3462)	0.0149 (0.0016‐0.1222)
RMM	8	0.0139 (0.0032‐0.0282)	0.0181 (0.0043‐0.0334)	0.0063 (0.0006‐0.0114)

HCs, healthy controls; NDMM, newly diagnosed multiple myeloma patients; RMM, relapsed/refractory multiple myeloma patients.

Among 37 NDMM patients, the percentage of iNKT cells was significantly lower in the patients with Stage C bone disease than those with Stage A/B bone disease and HCs (median 0.04% vs 0.07% and 0.09%, *P *=* *.042 and *P *<* *.001, respectively; Figure 1D). We analysed the serum level of biochemical markers of bone formation, such as OCN and PINP, bone destruction, such as CTX. The results suggested that the percentage of iNKT cells was strongly interrelated with the serum level of CTX (*r *=* *−.446, *P *=* *.006; Figure 1E). We have confirmed that OBPs and OCPs are sensitive for early diagnosing and monitoring bone disease of MM patients previously.^26^ The results indicated that the percentage of iNKT cells was significantly correlated with the population of OCPs (*r *=* *−.413, *P *=* *.011; Figure 1F). However, there were no significant differences between the level of iNKT cells percentage and the level of osteoblast precursors (OBPs), bone formation markers (OCN and PINP). According to the percentages of CD4^+^CD8^−^, CD4^−^CD8^+^ and DN iNKT cell subsets of T cells in the MM patients with Stage A/B or C bone disease (Table 4), the percentage of CD4^−^CD8^+^ iNKT cells was significantly lower in the patients with Stage C bone disease than those with Stage A/B bone disease and HCs (*P *=* *.029 and *P *<* *.001, respectively; Figure 1G). These results suggested that the quantity of iNKT cells which may be mainly CD4^−^CD8^+^ iNKT cells related to the state of bone destruction, especially osteoclastogenesis.

**Table 4:** The percentage of iNKT cells subsets in the T cell pool of newly diagnosed MM patients with Stage A/B or Stage C bone disease and healthy controls

Groups	Number	CD4^+^CD8^−^ iNKT (%)	CD4^−^CD8^−^ iNKT (%)	CD4^−^CD8^+^ iNKT (%)
Stage A/B	20	0.0197 (0.0052‐0.0725)	0.0411 (0.0088‐0.1121)	0.0071 (0.0012‐0.0306)
Stage C	17	0.0116 (0.0026‐0.0514)	0.0231 (0.0042‐0.0615)	0.0030 (0.0008‐0.0125)
HCs	23	0.0119 (0.0042‐0.0839)	0.0514 (0.0203‐0.5459)	0.0160 (0.0053‐0.0840)

HCs, healthy controls; MM, multiple myeloma.

### 3.2 The level of IFN‐γ produced by iNKT cells was impaired and significantly associated with bone disease in NDMM patients

**Figure 2:**
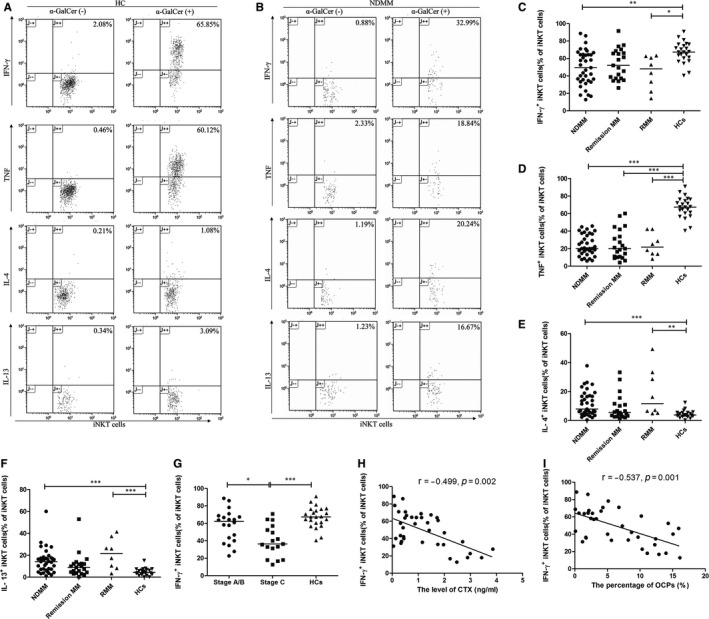
The level of IFN‐γ produced by iNKT cells was impaired and significantly associated with bone disease in newly diagnosed multiple myeloma (NDMM) patients. A,B, The expression of Th1 cytokines (IFN‐γ, TNF) and Th2 cytokines (IL‐4, IL‐13) by iNKT cells in healthy controls (HCs) and NDMM patients were examined by flow cytometry after stimulation with α‐GalCer. The percentages of IFN‐γ^+^ (C), TNF ^+^ (D) iNKT cells were significantly lower in NDMM and relapsed/refractory MM (RMM) patients than in HCs. The percentages of IL‐4^+^ (E) and IL‐13^+^ (F) iNKT cells were significantly higher in NDMM and RMM patients than in HCs. G, The percentages of IFN‐γ^+^ iNKT cells were strongly lower in NDMM patients with Stage C bone disease than with Stage A/B bone disease. H, The percentages of IFN‐γ^+^ iNKT cells was significantly correlated with the level of carboxy‐terminal cross‐linking telopeptide of type I collagen (CTX) respectively by Spearman's correlation coefficient. I, The percentages of IFN‐γ^+^ iNKT cells were significantly correlated with the percentages of osteoclast precursors (OCPs) respectively by Spearman's correlation coefficient. (Medians of each group were compared using Kruskal‐Wallis test followed by all pairwise multiple comparisons; *P <* *.05; **P <* *.01; ***P <* *.001)

We examined the expression of Th1 cytokines (IFN‐γ, TNF) and Th2 cytokines (IL‐4, IL‐13) by iNKT cells by flow cytometry after stimulation with α‐GalCer (Figure 2A and B). The levels of IFN‐γ and TNF in iNKT cells were significantly lower in NDMM and RMM patients than in HCs (median 49.43% and 48.13% vs 67.39%, *P *=* *.002 and *P *=* *.011, respectively; median 20.00% and 21.59% vs 67.39%, *P *<* *.001 and *P *<* *.001, respectively; Figure 2C, D). In addition, NDMM and RMM patients had significantly higher percentages of IL‐4^+^ and IL‐13^+^ iNKT cells than HCs (median 7.89% and 11.68% vs 3.77%, *P *=* *.001 and *P *=* *.004, respectively; median 14.14% and 21.61% vs 4.24%, *P *<* *.001 and *P *=* *.001, respectively; Figure 2E, F).

Among 37 NDMM patients, the level of IFN‐γ in iNKT cells was strongly lower in NDMM patients with advanced bone disease (Stage C) than with bone disease (Stage A/B; median 36.36% vs 62.38%, *P *=* *.030; Figure 2G), which were negatively correlated with the serum level of CTX (*r *=* *−.499, *P *=* *.002; Figure 2H) and the population of OCPs (*r *=* *−.537, *P *=* *.001; Figure 2I). However, there were no significant differences between the levels of TNF^+^, IL‐4^+^, IL‐13^+^ and OBPs, OCN and PINP. These results indicated that the level of IFN‐γ produced by iNKT cells was related with osteoclastogenesis in MM.

### 3.3 Deficiency in proliferative responses of iNKT cells on α‐GalCer and impaired regulation of osteoclastogenesis by iNKT cells in NDMM patients

**Figure 3:**
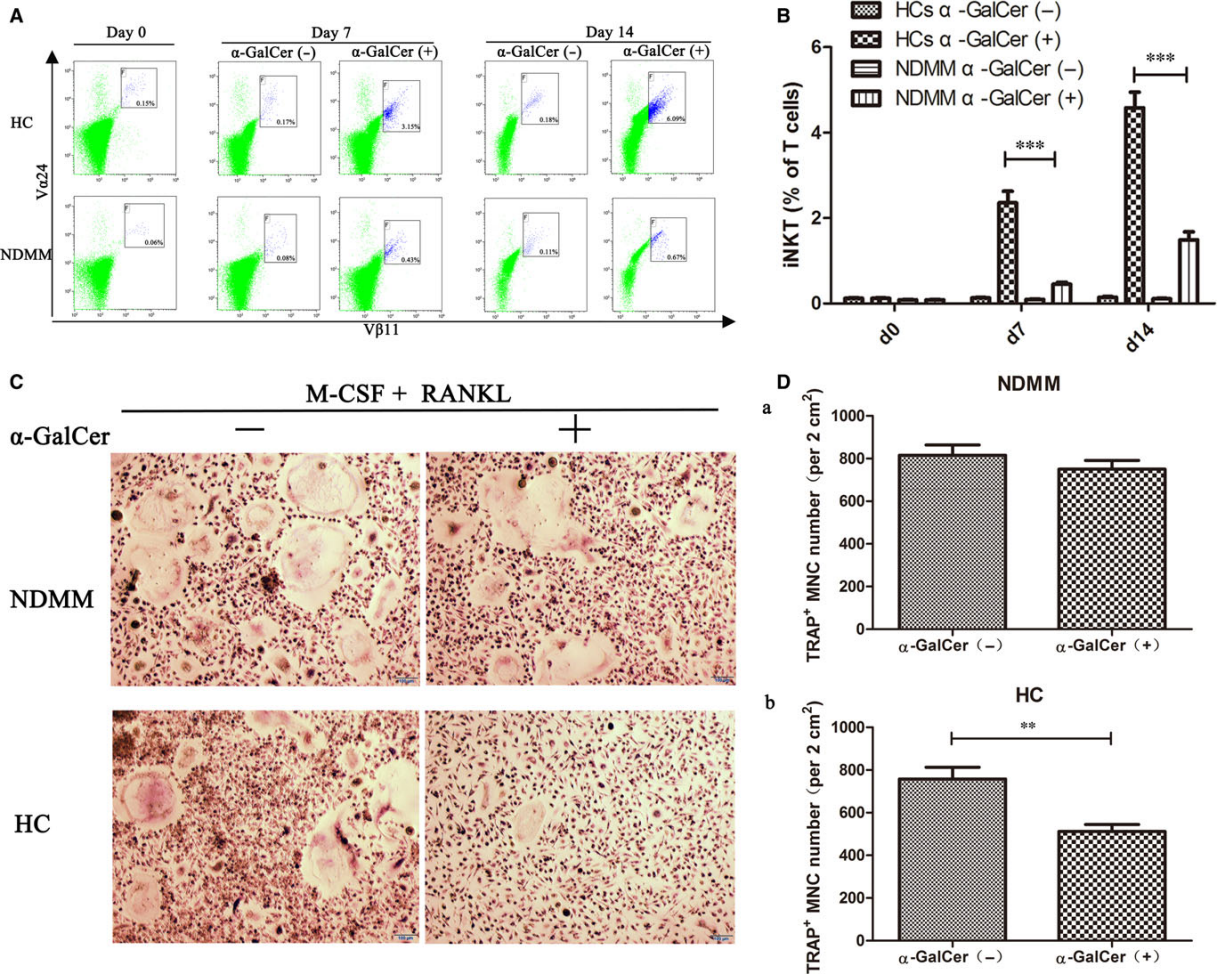
Deficiency in proliferative responses of iNKT cells on α‐GalCer and impaired in regulation of osteoclastogenesis by iNKT cells in newly diagnosed multiple myeloma (NDMM) patients. A, The proliferative responses of iNKT cells stimulated by α‐GalCer in NDMM patients and healthy controls (HCs) at day 0, day 7 and day 14 were examined by flow cytometry. B, The percentages of iNKT cells in HCs were markedly higher than in NDMM patients after stimulated by α‐GalCer at days 7 and 14. C, Peripheral blood mononuclear cells (PBMCs) from 12 NDMM patients and 12 HCs were cultured with RANKL and macrophage colony‐stimulating factor (M‐CSF) in the presence of α‐GalCer or 0.1% DMSO (as control) and then stained for tartrate‐resistant acid phosphate (TRAP) as described in Section 2. Representative TRAP ^+^ multinucleated cells (MNC) from a NDMM patient and a HC. Original magnification × 100 (Bar = 100 μm). D(a), About the number of TRAP ^+^ MNCs, there were no significant differences between α‐GalCer‐stimulated cultures and DMSO control cultures in NDMM patients. (b) In HCs, the number of TRAP ^+^ MNCs was significantly decreased in α‐GalCer‐stimulated cultures compared with DMSO control cultures. (Mean ± SEM of each group were compared using Student's *t* test; *P <* *.05, **P <* *.01, ***P <* *.001)

We examined the proliferative responses of iNKT cells on α‐GalCer in 12 NDMM patients and 12 HCs at day 0, day 7 and day 14 by flow cytometry (Figure 3A). For the percentages of iNKT cells in the T cells, HCs were markedly higher than in NDMM patients after stimulated by α‐GalCer at day 7 (mean ± SEM 2.359 ± 0.267% vs 0.451 ± 0.046%, *P *<* *.001) and at day 14 (mean ± SEM 4.584 ± 0.362% vs 1.490 ± 0.188%, *P *<* *.001; Figure 3B). These results indicated that the proliferative effects of iNKT cells on α‐GalCer were impaired in NDMM patients.

To explore whether iNKT cells regulate osteoclast formation, PBMCs from 12 NDMM patients and 12 HCs were cultured with RANKL and M‐CSF in the presence of α‐GalCer or 0.1% DMSO (as control) and then stained for TRAP (Figure 3C). In HCs, the number of TRAP^+^ multinucleated cells (MNC) was significantly decreased in α‐GalCer‐stimulated cultures compared with DMSO control cultures (mean ± SEM 512 ± 32 cells per 2 cm^2^ vs 758 ± 55 cells per 2 cm^2^; *P *=* *.001; Figure 3D(b)). However, there were no significant differences between α‐GalCer‐stimulated cultures and DMSO control cultures in NDMM patients (mean ± SEM 750 ± 41 cells per 2 cm^2^ vs 816 ± 48 cells per 2 cm^2^; *P *=* *.310; Figure 3D(a)). The results suggested that the regulation of osteoclastogenesis by iNKT cells in vitro was impaired in NDMM patients.

### 3.4 Inhibition of osteoclastogenesis by iNKT cells via IFN‐γ production

**Figure 4:**
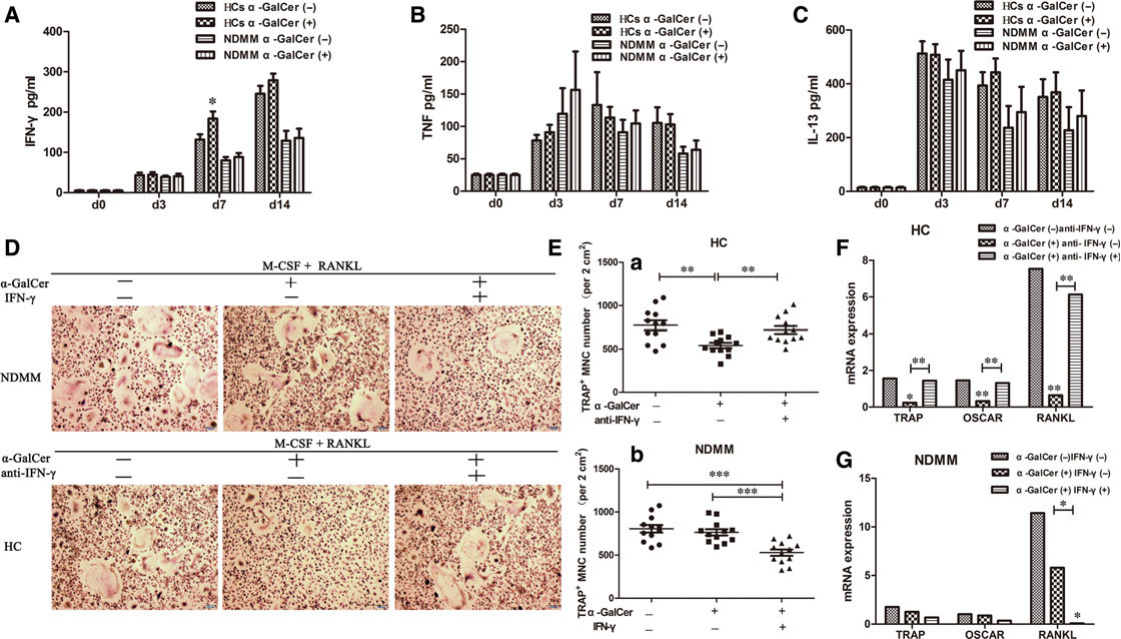
Inhibition of osteoclastogenesis by iNKT cells via IFN‐γ production. A, In healthy controls (HCs), the level of IFN‐γ in osteoclasts (OCs) culture supernatants at day 7 and day 14 markedly increased in α‐GalCer‐stimulated cultures compared with DMSO control cultures. The differences at day 7 were significant, but at day 14 were no significant. About the level of TNF (B) and IL‐13 (C) in OCs culture supernatants, no significant differences were found between α‐GalCer‐stimulated cultures and DMSO control cultures at day 0, day 3, day 7 and day 14 in NDMM patients and HCs. Mean ± SEM between the experimental samples were compared using Student's *t* test. D, Representative tartrate‐resistant acid phosphate (TRAP)‐positive multinucleated cells (MNC) from a NDMM patient in the presence or absence of recombinant IFN‐γ or α‐GalCer and a HC in the presence or absence of anti‐IFN‐γ or α‐GalCer. Original magnification × 100 (Bar = 100 μm). E(a), The number of TRAP ^+^ MNCs was significantly increased in the presence of anti‐IFN‐γ and α‐GalCer cultures compared with the presence of α‐GalCer cultures (b) The number of TRAP ^+^ MNCs was significantly reduced in the presence of IFN‐γ and α‐GalCer cultures compared with the presence of α‐GalCer cultures. Mean ± SEM of each group were compared using one‐way ANOVA analysis. F, The mRNA expression of osteoclast‐associated genes, such as TRAP, osteoclast‐associated receptor (OSCAR) and RANKL was significantly improved in the presence of anti‐IFN‐γ and α‐GalCer cultures compared with the presence of α‐GalCer cultures. Medians of each group were compared using Kruskal‐Wallis test followed by all pairwise multiple comparisons. G**,** The mRNA expression of RANKL was significantly decreased in the presence of IFN‐γ and α‐GalCer cultures compared with the presence of α‐GalCer cultures. Medians of each group were compared using Kruskal‐Wallis test followed by all pairwise multiple comparisons (*P <* *.05, **P <* *.01, ***P <* *.001)

To examine whether inhibition of osteoclastogenesis is regulated by cytokines produced by iNKT cells, we measured the level of IFN‐γ, TNF, IL‐4 or IL‐13 in OCs culture supernatants from the 12 NDMM patients and 12 HCs by ELISA kit at day 0, day 3, day 7 and day 14. In HCs, the level of IFN‐γ at day 7 and day 14 markedly increased in α‐GalCer‐stimulated cultures compared with DMSO control cultures. The differences at day 7 were significant (mean ± SEM 183.73 ± 17.76 vs 131.97 ± 12.83; *P *=* *.031), but at day 14 were no significant (mean ± SEM 279.07 ± 16.44 vs 245.34 ± 19.83, *P *=* *.209; Figure 4A). However, no significant differences were found between α‐GalCer‐stimulated cultures and DMSO control cultures about the levels of TNF and IL‐13 at day 0, day 3, day 7 and day 14. In addition, for NDMM patients, the levels of IFN‐γ, TNF and IL‐13 have no differences between α‐GalCer‐stimulated cultures and DMSO control cultures (Figure 4A‐C). The level of IL‐4 in OCs culture supernatants was not detected in both HCs and in NDMM patients. The results suggest that inhibition of osteoclastogenesis may be mediated by IFN‐γ production of iNKT cells.

To confirm IFN‐γ produced by iNKT cells inhibit osteoclastogenesis, PBMCs from 12 HCs were cultured with RANKL and M‐CSF for 14 days with or without anti‐IFN‐γ or α‐GalCer and then stained for TRAP (Figure 4D). The number of TRAP^+^ MNCs was significantly increased in the presence of anti‐IFN‐γ and α‐GalCer cultures compared with the presence of α‐GalCer cultures (mean ± SEM 719 ± 45 cells per 2 cm^2^ vs 542 ± 31 cells per 2 cm^2^; *P *=* *.009; Figure 4E(a)). In addition, the mRNA expression of osteoclast‐associated genes, such as TRAP, osteoclast‐associated receptor (OSCAR) and RANKL was significantly improved in the presence of anti‐IFN‐γ and α‐GalCer cultures compared with the presence of α‐GalCer cultures (Figure 4F).

Meanwhile, PBMCs from 20 NDMM patients were cultured as the same as HCs (Figure 4D). The number of TRAP^+^ MNCs was significantly reduced in the presence of IFN‐γ and α‐GalCer cultures compared with the presence of α‐GalCer cultures (mean ± SEM 529 ± 37 cells per 2 cm^2^ vs 765 ± 36 cells per 2 cm^2^; *P *<* *.001; Figure 4E(b)). In addition, the mRNA expression of RANKL was significantly decreased in the presence of IFN‐γ and α‐GalCer cultures compared with the presence of α‐GalCer cultures (Figure 4G).

## 4 DISCUSSION

Multiple myeloma (MM) is a malignancy of malignant plasma cells and occurs bone disease in 80% of patients owing to imbalance between osteoclasts and osteoblasts.^29^ How to cure the bone disease of MM patients is still a challenge. Recent studies indicated the immune system has an important role in the osteoblast and osteoclast activity.^10^ iNKT cells are a distinct subset of T cells that exhibit bridges connecting the innate immune system to the adaptive immune system and regulate development and function of monocytes and macrophages.^30^, ^31^ Osteoclasts development from monocytes‐macrophages plays a critical role in bone remodelling.^32^ However, the effect of iNKT on osteoclasts activity of myeloma bone disease remains unknown. Our study first investigated the role of iNKT cells in osteoclastogenesis and the underlying mechanism in MBD.

It was previously reported that iNKT cells are a small population of T lymphocytes derived from the thymus at the double positive selection (CD4^+^CD8^+^) stage of T cell development [33,34]. While a significant proportion of iNKT cells remain in the thymus, where they become long‐lived residents, other part of iNKT cells are often migrated to different tissues, like liver, spleen, bone marrow, peripheral blood and lymph nodes [35,36]. In healthy humans, the relative frequency of iNKT cells of BM is similar to that of PB [37]. The number of iNKT cells in bone marrow of MM patients was rarely reported. But, the group of Dhodapkar have provided evidence that iNKT cells from PB and BM are functionally deficient (secretion IFN‐γ) in MM patients [20]. iNKT cells from bone marrow or peripheral blood are homologous and their functions are similar. Furthermore, iNKT cells in peripheral blood are easy to be measured and used to estimate bone disease in MM patients. So, we choosed iNKT cell from peripheral blood for the experiment. By observing the distribution of iNKT cells in different MM patients, we found that the quantity of iNKT cells which primarily was CD4^−^CD8^+^ and DN iNKT cells were defective in NDMM and RMM patients, the production of Th1 cytokines (IFN‐γ and TNF) by iNKT cells also reduced and Th2 cytokines (IL‐4 and IL‐13) by iNKT cells increased in NDMM and RMM patients. Different previously researches provided evidence of iNKT cells being quantitatively and qualitatively defective in MM, subsequently hampering their antitumour effects,^33^ and they observed that advanced stages of MM were associated with the progressive loss of the ability of iNKT cells to secrete IFN‐γ. A comparative deficit of the CD4^−^ iNKT cell subset that is regarded as the most important for anticancer activities hampers their antitumour effects and leads to defective production of Th1 cytokines.^34^, ^35^ In addition, the percentages of iNKT cells (including CD4^−^CD8^+^ iNKT cells) and IFN‐γ^+^ iNKT cells were significantly lower in the patients with Stage C bone disease than those with Stage A/B bone disease and HCs. These results suggested that the quantity of iNKT cells which may be mainly CD4^−^CD8^+^ iNKT cells related to the state of bone destruction. Because the quantity of CD4^−^CD8^+^ iNKT cells is very small, the role of them cannot be observed alone. Therefore, our study focused on total iNKT cells.

It is well known that the serum level of OCN,^36^ PINP^37^ and CTX^38^ and the percentages of OBPs and OCPs are sensitive for early diagnosing and monitoring bone disease of MM patients previously.^25^, ^26^ Our results also suggested that the percentages of iNKT cells and IFN‐γ^+^ iNKT cells were strongly interrelated with the serum level of CTX and the population of OCPs. We deduce that the level of iNKT cells frequency and production function of IFN‐γ relate to the state of bone destruction, especially osteoclastogenesis. In addition, some studies concluded that immune cells such as T cells express RANKL, but T cells also resist osteoclastogenesis by producing interferon‐γ (IFN‐γ), which counterbalance the action of RANKL.^4^, ^7^, ^8^ iNKT cells acting as a special T cell may be involved in bone destruction, especially osteoclastogenesis.

To investigate the role of iNKT cells in osteoclastogenesis further, we used co‐culture to confirm that the proliferative responses of iNKT cells on α‐GalCer were disturbed and the regulation of osteoclastogenesis by iNKT cells was impaired in NDMM patients and that this impairment was associated with iNKT cells deficiency. Furthermore, we measuring the level of IFN‐γ, TNF, IL‐4 or IL‐13 in OCs culture supernatants from NDMM patients and HCs by ELISA kit proved that inhibition of osteoclastogenesis may be regulated by IFN‐γ production in iNKT cells. Moreover, we performed with anti‐IFN‐γ blocking antibodies and recombinant IFN‐γ investigated that inhibition of osteoclastogenesis by iNKT cells was regulated by IFN‐γ production, which down‐regulated osteoclast‐associated genes, such as RANKL, OSCAR and TRAP.

Furthermore, the group of Jin et al also conducted negative regulation of osteoclastogenesis by NKT cells is impaired in Rheumatoid Arthritis patients *in intro* and inhibition of osteoclastogenesis by NKT cells was predominantly mediated by IFN‐γ signalling in vivo.^21^

But Hu et al used iNKT cell‐deficient and wild‐type mice to demonstrate that selective activation of iNKT cells by α‐GalCer causes myeloid cell egress, enhances OC progenitor and precursor development, modifies the intramedullary kinetics of mature OCs and enhances their resorptive activity. OC progenitor activity is positively regulated by TNF‐α and negatively regulated by IFN‐γ, but is IL‐4 and IL‐17 independent.^22^ However, our study indicated that the percentage of iNKT cells was significantly correlated with the population of OC progenitors. As a result of the experiment of Hu et al was carried out only in normal mice without disease and our study was lack of mice experiment in vivo, further experiments need to be conducted.

In addition, Spanoudakis et al found that higher levels of RANKL expressed by iNKT cells in peripheral blood and especially BM of MM patients as part of a myeloma‐specific dysfunctional iNKT cell phenotype that could contribute to osteoclast activation and bone destruction as well as tumour immune evasion.^39^ Owing to the experiment of Spanoudakis et al did not involve mechanism research and the lack of related researches about iNKT in myeloma bone disease, further experiments in vitro or *vivo* need to be performed.

Our study presented here emphasized the potential role of α‐GalCer‐stimulated iNKT cells in regulation of osteoclastogenesis and inhibition of bone destruction. However, this function was impaired in myeloma bone disease patients as a result of iNKT cell dysfunction. Further studies about the ways of repairing iNKT cell deficiency and providing important evidence of promising therapeutic strategy in myeloma bone disease patients need to be conducted.

## AUTHORS’ CONTRIBUTIONS

Rong Fu designed the research and revised the manuscript. Fengjuan Jiang, Hui Liu and Zhaoyun Liu performed the experiments, analysed the data and wrote the article. Siyang Yan, Jin Chen, Qing Shao, Lijuan Li, Jia Song, Guojin Wang and Zonghong Shao contributed to the experimental work and the collection of patients’ features. All authors read and approved the final manuscript.

## CONFLICT OF INTEREST

The authors confirm that there are no conflict of interests.

## References

[1] Rajkumar SV , Buadi F . Multiple myeloma: new staging systems for diagnosis, prognosis and response evaluation. Best Pract Res Clin Haematol. 2007;20:665‐680. 18070712 10.1016/j.beha.2007.10.002

[2] Oranger A , Carbone C , Izzo M , Grano M . Cellular mechanisms of multiple myeloma bone disease. Clin Dev Immunol. 2013;2013:289458. 23818912 10.1155/2013/289458 PMC3681224

[3] Boyle WJ , Simonet WS , Lacey DL . Osteoclast differentiation and activation. Nature. 2003;423:337‐342. 12748652 10.1038/nature01658

[4] Giuliani N , Colla S , Sala R , et al. Human myeloma cells stimulate the receptor activator of nuclear factor‐kappa B ligand (RANKL) in T lymphocytes: a potential role in multiple myeloma bone disease. Blood. 2002;100:4615‐4621. 12393684 10.1182/blood-2002-04-1121

[5] Colucci S , Brunetti G , Mori G , et al. Soluble decoy receptor 3 modulates the survival and formation of osteoclasts from multiple myeloma bone disease patients. Leukemia. 2009;23:2139‐2146. 19587706 10.1038/leu.2009.136

[6] Brunetti G , Rizzi R , Oranger A , et al. LIGHT/TNFSF14 increases osteoclastogenesis and decreases osteoblastogenesis in multiple myeloma‐bone disease. Oncotarget. 2014;5:12950‐12967. 25460501 10.18632/oncotarget.2633 PMC4350341

[7] Takayanagi H , Ogasawara K , Hida S , et al. T‐cell‐mediated regulation of osteoclastogenesis by signalling cross‐talk between RANKL and IFN‐gamma. Nature. 2000;408:600‐605. 11117749 10.1038/35046102

[8] Colucci S , Brunetti G , Rizzi R , et al. T cells support osteoclastogenesis in an in vitro model derived from human multiple myeloma bone disease: the role of the OPG/TRAIL interaction. Blood. 2004;104:3722‐3730. 15308561 10.1182/blood-2004-02-0474

[9] Sato K , Suematsu A , Okamoto K , et al. Th17 functions as an osteoclastogenic helper T cell subset that links T cell activation and bone destruction. J Exp Med. 2006;203:2673‐2682. 17088434 10.1084/jem.20061775 PMC2118166

[10] Takayanagi H . Osteoimmunology: shared mechanisms and crosstalk between the immune and bone systems. Nat Rev Immuno. 2007;7:292‐304. 10.1038/nri2062 17380158

[11] Lubberts E , Joosten LA , Chabaud M , et al. IL‐4 gene therapy for collagen arthritis suppresses synovial IL‐17 and osteoprotegerin ligand and prevents bone erosion. J Clin Invest. 2000;105:1697‐1710. 10862785 10.1172/JCI7739 PMC378501

[12] Cerundolo V , Kronenberg M . The role of invariant NKT cells at the interface of innate and adaptive immunity. Semin Immmunol. 2010;22:59‐60. 10.1016/j.smim.2010.01.002 20172739

[13] Benlagha K , Bendelac A . CD1d‐restricted mouse V alpha 14 and human V alpha 24 T cells: lymphocytes of innate immunity. Semin Immunol. 2000;12:537‐542. 11145859 10.1006/smim.2000.0276

[14] Kobayashi E , Motoki K , Uchida T , et al. KRN7000, a novel immunomodulator, and its antitumor activities. Oncol Res. 1995;7:529‐534. 8866665

[15] Kawano T , Cui J , Koezuka Y , et al. CD1d‐restricted and TCR‐mediated activation of valpha14 NKT cells by glycosylceramides. Science. 1997;278:1626‐1629. 9374463 10.1126/science.278.5343.1626

[16] Van Kaer L . alpha‐Galactosylceramide therapy for autoimmune diseases: prospects and obstacles. Nat Rev Immunol. 2005;5:31‐42. 15630427 10.1038/nri1531

[17] Dhodapkar MV , Richter J . Harnessing natural killer T (NKT) cells in human myeloma: progress and challenges. Clin Immunol. 2011;140:160‐166. 21233022 10.1016/j.clim.2010.12.010 PMC3224820

[18] Chan AC , Neeson P , Leeansyah E , et al. Natural killer T cell defects in multiple myeloma and the impact of lenalidomide therapy. Clin Exp Immunol. 2014;175:49‐58. 24032527 10.1111/cei.12196 PMC3898554

[19] Richter J , Neparidze N , Zhang L , et al. Clinical regressions and broad immune activation following combination therapy targeting human NKT cells in myeloma. Blood. 2013;121:423‐430. 23100308 10.1182/blood-2012-06-435503 PMC3548165

[20] Dhodapkar MV , Geller MD , Chang DH , et al. A reversible defect in natural killer t cell function characterizes the progression of premalignant to malignant multiple myeloma. J Exp Med. 2003;197:1667‐1676. 12796469 10.1084/jem.20021650 PMC2193955

[21] Jin H , Kee S , Cho Y , et al. Dysregulated osteoclastogenesis is related to natural killer T cell dysfunction in rheumatoid arthritis. Arthritis Rheumatol. 2015;67:2639‐2650. 26097058 10.1002/art.39244

[22] Hu M , Bassett JH , Danks L , et al. Activated invariant NKT cells regulate osteoclast development and function. J Immunol. 2011;186:2910‐2917. 21278350 10.4049/jimmunol.1002353

[23] Terpos E , de la Fuente J , Szydlo R , et al. Tartrate‐resistant acid phosphatase isoform 5b: a novel serum marker for monitoring bone disease in multiple myeloma. Int J Cancer. 2003;106:455‐457. 12845688 10.1002/ijc.11247

[24] Terpos E , Christoulas D , Kastritis E , et al. The combination of lenalidomide and dexamethasone reduces bone resorption in responding patients with relapsed/refractory multiple myeloma but has no effect on bone formation: final results on 205 patients of the Greek myeloma study group. Am J Hematol. 2014;89:34‐40. 23983166 10.1002/ajh.23577

[25] D'Amelio P , Tamone C , Sassi F , et al. Teriparatide increases the maturation of circulating osteoblast precursors. Osteoporos Int. 2012;23:1245‐1253. 21617993 10.1007/s00198-011-1666-2

[26] Fu R , Peng F , Liu H , et al. Clinical significance of osteoblast precursors and osteoclast precursors in earlier diagnosis and monitoring of myeloma bone disease. Ann Hematol. 2016;95:1099‐1106. 27118542 10.1007/s00277-016-2657-3

[27] Sandberg JK , Bhardwaj N , Nixon DF . Dominant effector memory characteristics, capacity for dynamic adaptive expansion, and sex bias in the innate Valpha24 NKT cell compartment. Eur J Immunol. 2003;33:588‐596. 12616479 10.1002/eji.200323707

[28] Liu H , Peng F , Liu Z , et al. CYR61/CCN1 stimulates proliferation and differentiation of osteoblasts in vitro and contributes to bone remodeling in vivo in myeloma bone disease. Int J Oncol. 2017;50:631‐639. 28035364 10.3892/ijo.2016.3815

[29] Giuliani N , Rizzoli V , Roodman GD . Multiple myeloma bone disease: Pathophysiology of osteoblast inhibition. Blood. 2006;108:3992‐3996. 16917004 10.1182/blood-2006-05-026112

[30] Nieuwenhuis EE , Matsumoto T , Exley M , et al. CD1d‐dependent macrophage‐mediated clearance of *Pseudomonas aeruginosa* from lung. Nat Med. 2002;8:588‐593. 12042809 10.1038/nm0602-588

[31] Lorenzo J , Horowitz M , Choi Y . Osteoimmunology: interactions of the bone and immune system. Endocr Rev. 2008;29:403‐440. 18451259 10.1210/er.2007-0038 PMC2528852

[32] Fogg DK , Sibon C , Miled C , et al. A clonogenic bone marrow progenitor specific for macrophages and dendritic cells. Science. 2006;311:83‐87. 16322423 10.1126/science.1117729

[33] Dhodapkar MV , Geller MD , Chang DH , et al. A reversible defect in natural killer T cell function characterizes the progression of premalignant to malignant multiple myeloma. J Exp Med. 2003;197:1667‐1676. 12796469 10.1084/jem.20021650 PMC2193955

[34] Chan AC , Leeansyah E , Cochrane A , et al. Ex‐vivo analysis of human natural killer T cells demonstrates heterogeneity between tissues and within established CD4(+) and CD4(‐) subsets. Clin Exp Immunol. 2013;172:129‐137. 23480193 10.1111/cei.12045 PMC3719939

[35] Crowe NY , Coquet JM , Berzins SP , et al. Differential antitumor immunity mediated by NKT cell subsets in vivo. J Exp Med. 2005;202:1279‐1288. 16275765 10.1084/jem.20050953 PMC1459911

[36] Woitge HW , Horn E , Keck AV , et al. Biochemical markers of bone formation in patients with plasma cell dyscrasias and benign osteoporosis. Clin Chem. 2001;47:686‐693. 11274019

[37] Kowalska M , Druzd‐Sitek A , Fuksiewicz M , et al. Procollagen I amino‐terminal propeptide as a potential marker for multiple myeloma. Clin Biochem. 2010;43:604‐608. 20045402 10.1016/j.clinbiochem.2009.12.018

[38] Lund T , Abildgaard N , Andersen TL , et al. Multiple myeloma: changes in serum C‐terminal telopeptide of collagen type I and bone‐specific alkaline phosphatase can be used in daily practice to detect imminent osteolysis. Eur J Haematol. 2010;84:412‐420. 20070853 10.1111/j.1600-0609.2010.01417.x PMC2871171

[39] Spanoudakis E , Papoutselis M , Terpos E , et al. Overexpression of RANKL by invariant NKT cells enriched in the bone marrow of patients with multiple myeloma. Blood Cancer J. 2016;6:e500. 27834938 10.1038/bcj.2016.108 PMC5148055

